# Supplementary material for: Seasonal Chemical Evaluation of Miconia chamissois Naudin from Brazilian Savanna
Source: Molecules. 2022 Feb 8;27(3):1120. doi: 10.3390/molecules27031120 (PMC8838837; doi:10.3390/molecules27031120)
Supplement: Supplementary file 1 [file molecules-27-01120-s001.zip › molecules-1555558-supplementary.pdf]

Supplementary material

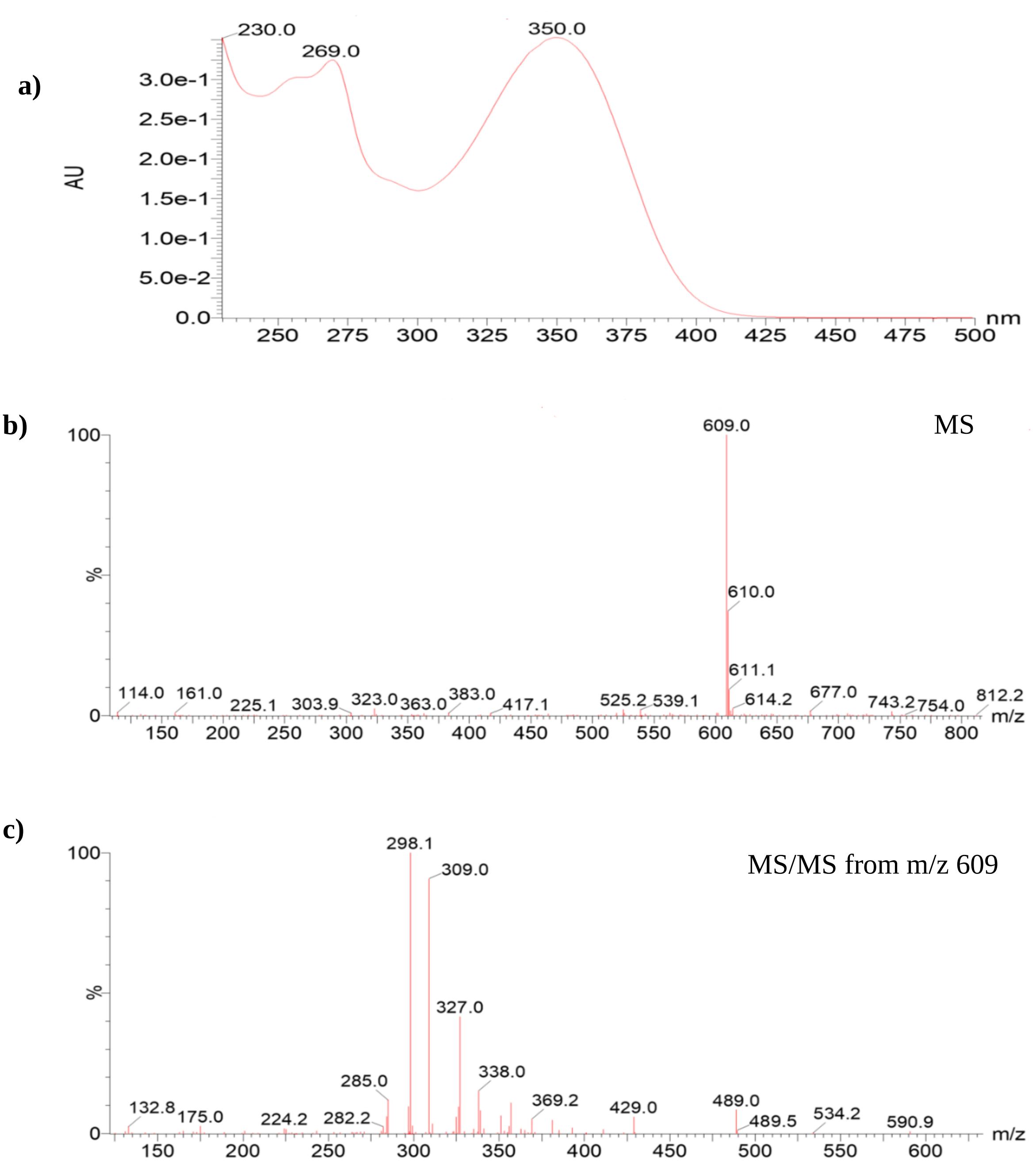

Figure S1: UHPLC-UV/MS/MS data of peak 4. a) UV-Vis spectrum; b) full scan negative ion electrospray mass spectrum c) negative ion electrospray MS/MS spectrum, with collision energy of 40V. This suggested a mixed O, C-glycoside of luteolin.

a)

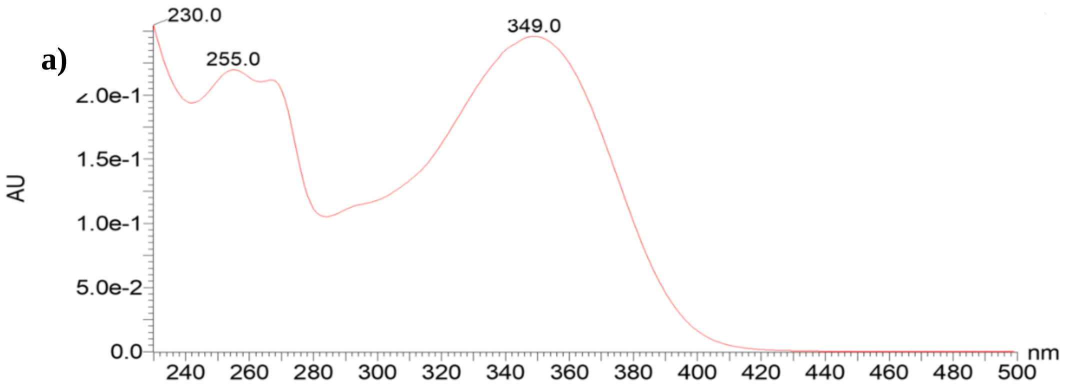

b)

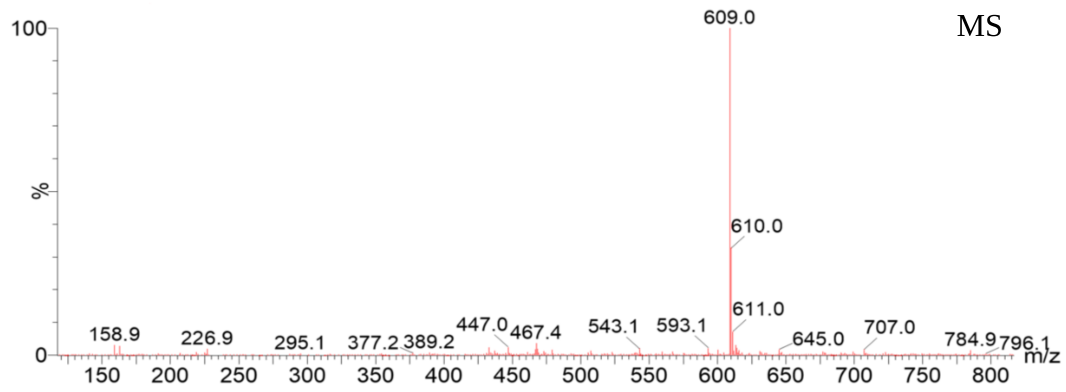

c)

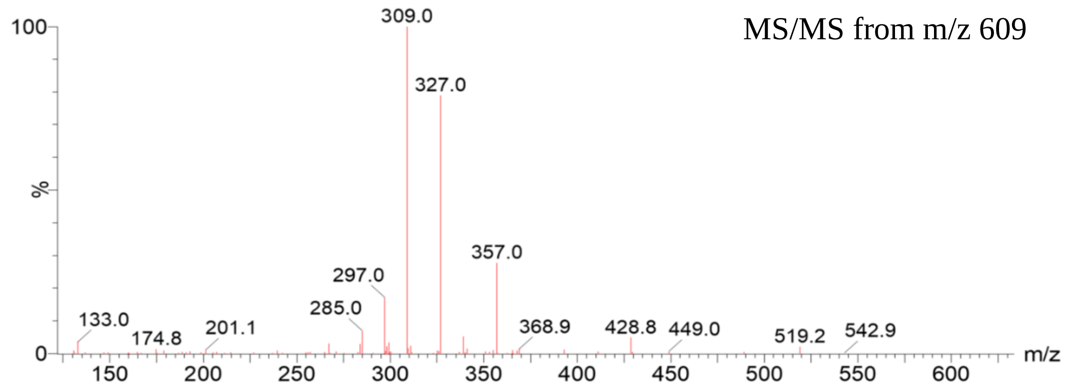

Figure S2: UHPLC-UV/MS/MS data of peak 5. a) UV-Vis spectrum; b) full scan negative ion electrospray mass spectrum; c) negative ion electrospray MS/MS spectrum, with collision energy of 40V. This suggested a mixed O, C-glycoside of luteolin.

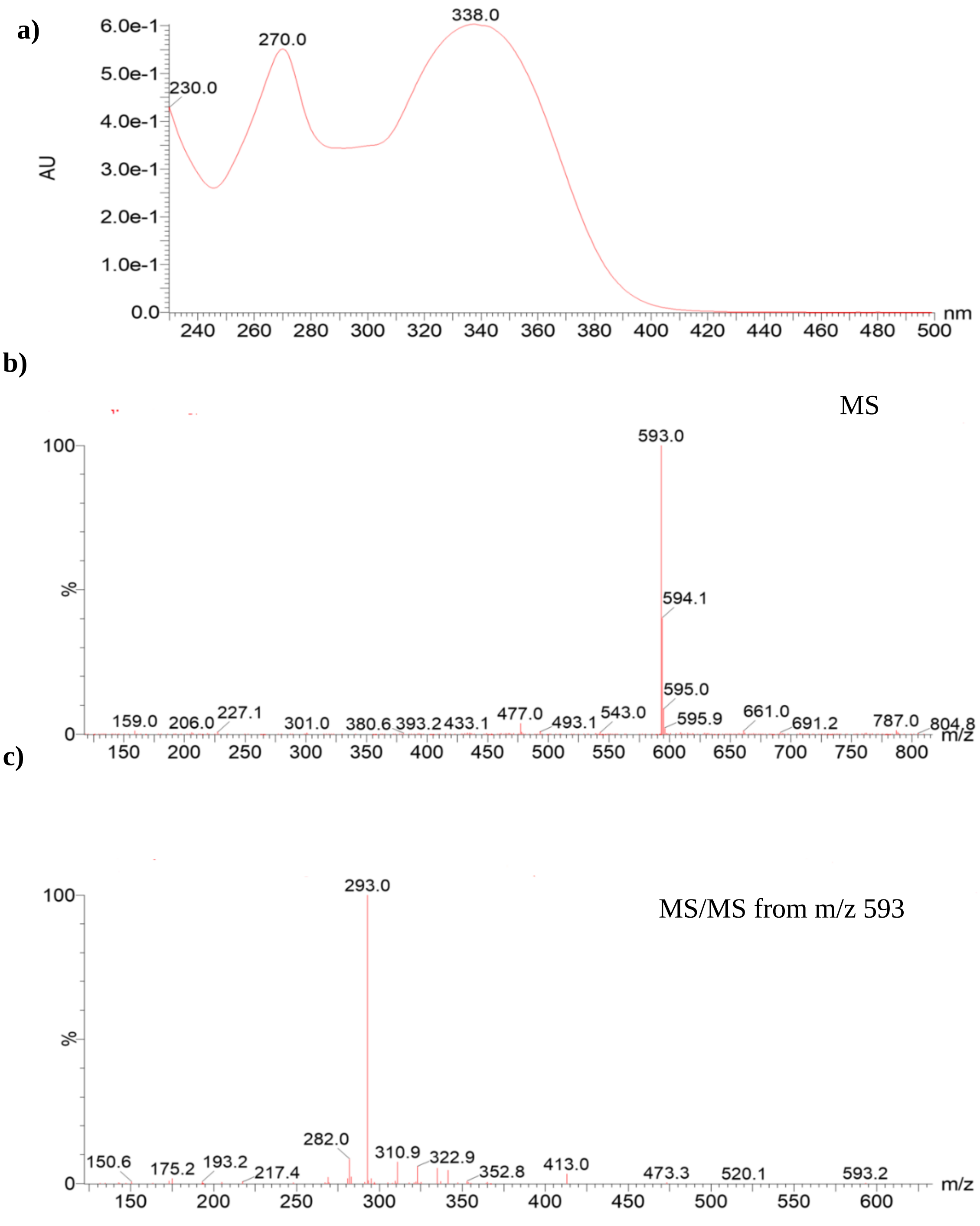

Figure S3: UHPLC-UV/MS/MS data of peak 6. a) UV-Vis spectrum ; b) full scan negative ion electrospray mass spectrum; c) negative ion electrospray MS/MS spectrum, with collision energy of 40V. This suggested a mixed O,C-glycoside of apigenin.

a)

b)

c)

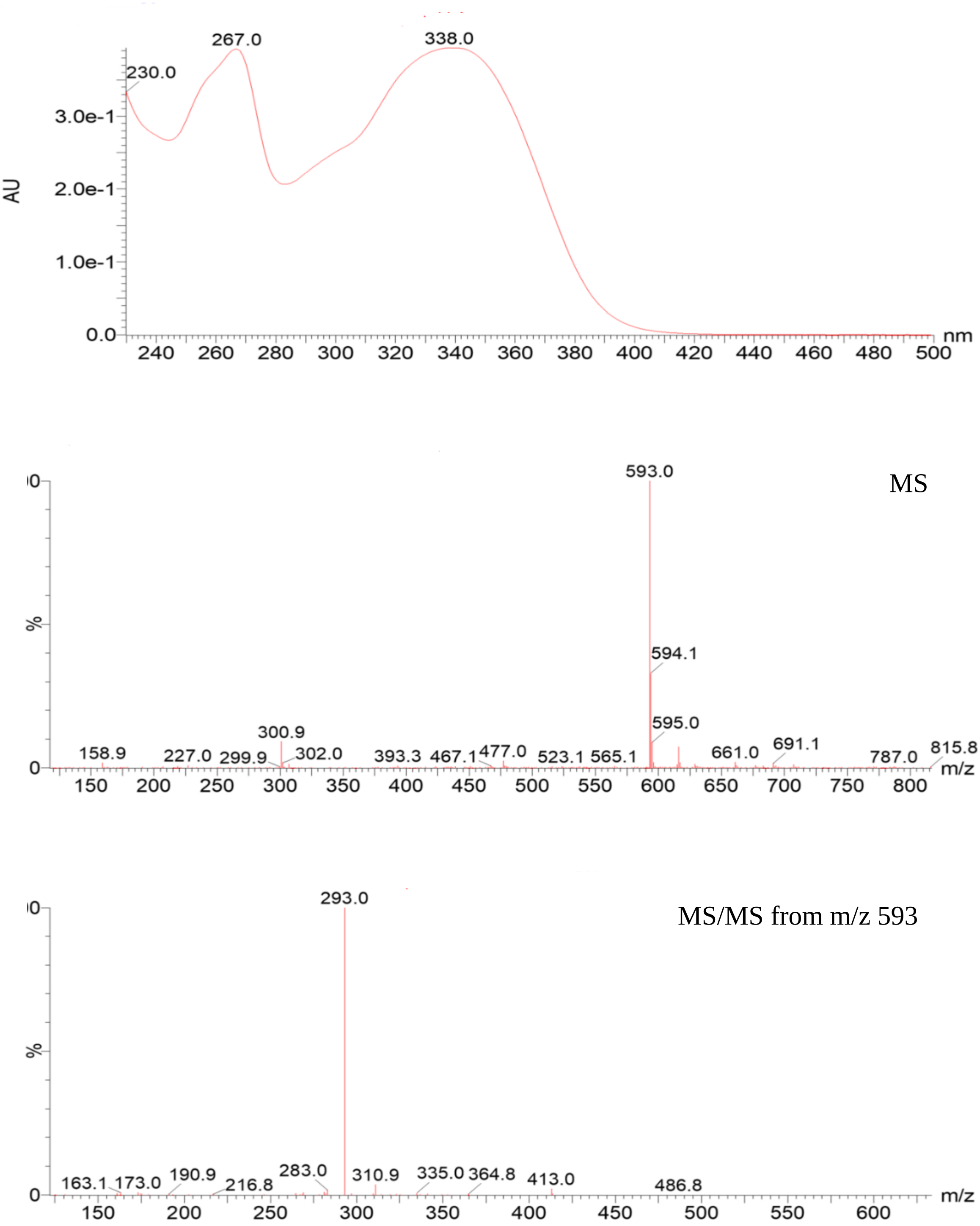

Figure S4: UHPLC-UV/MS/MS data of peak 7. a) UV-Vis spectrum; b) full scan negative ion mass spectrum; c) negative ion electrospray MS/MS spectrum, with collision energy of 40V. This suggested a mixed O,C-glycoside of apigenin.

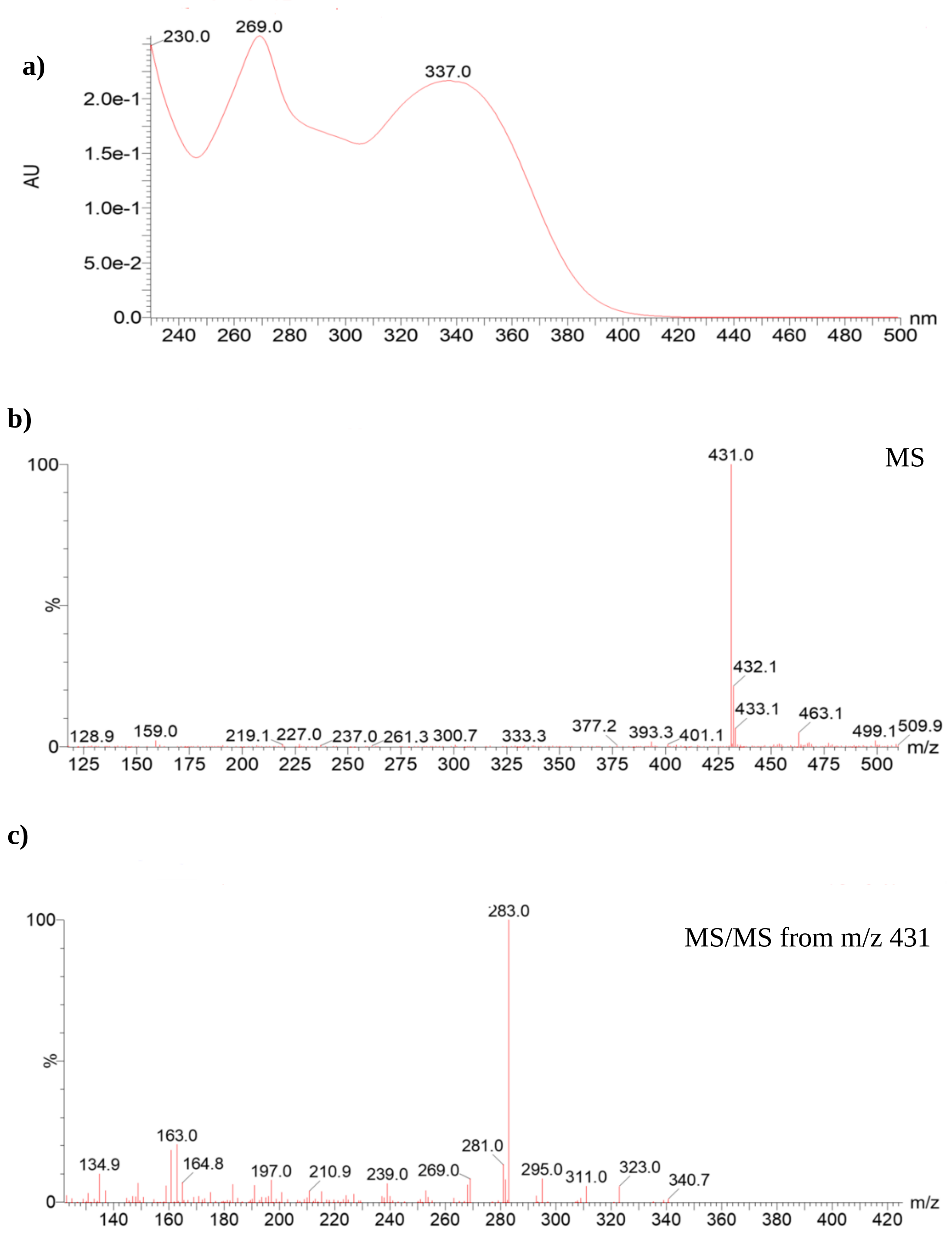

Figure S5: UHPLC-UV/MS/MS data of peak 8. a) UV-Vis spectrum; b) full scan negative ion electrospray mass spectrum; c) negative ion electrospray MS/MS spectrum, with collision energy of 40V. This matched isovitexin.

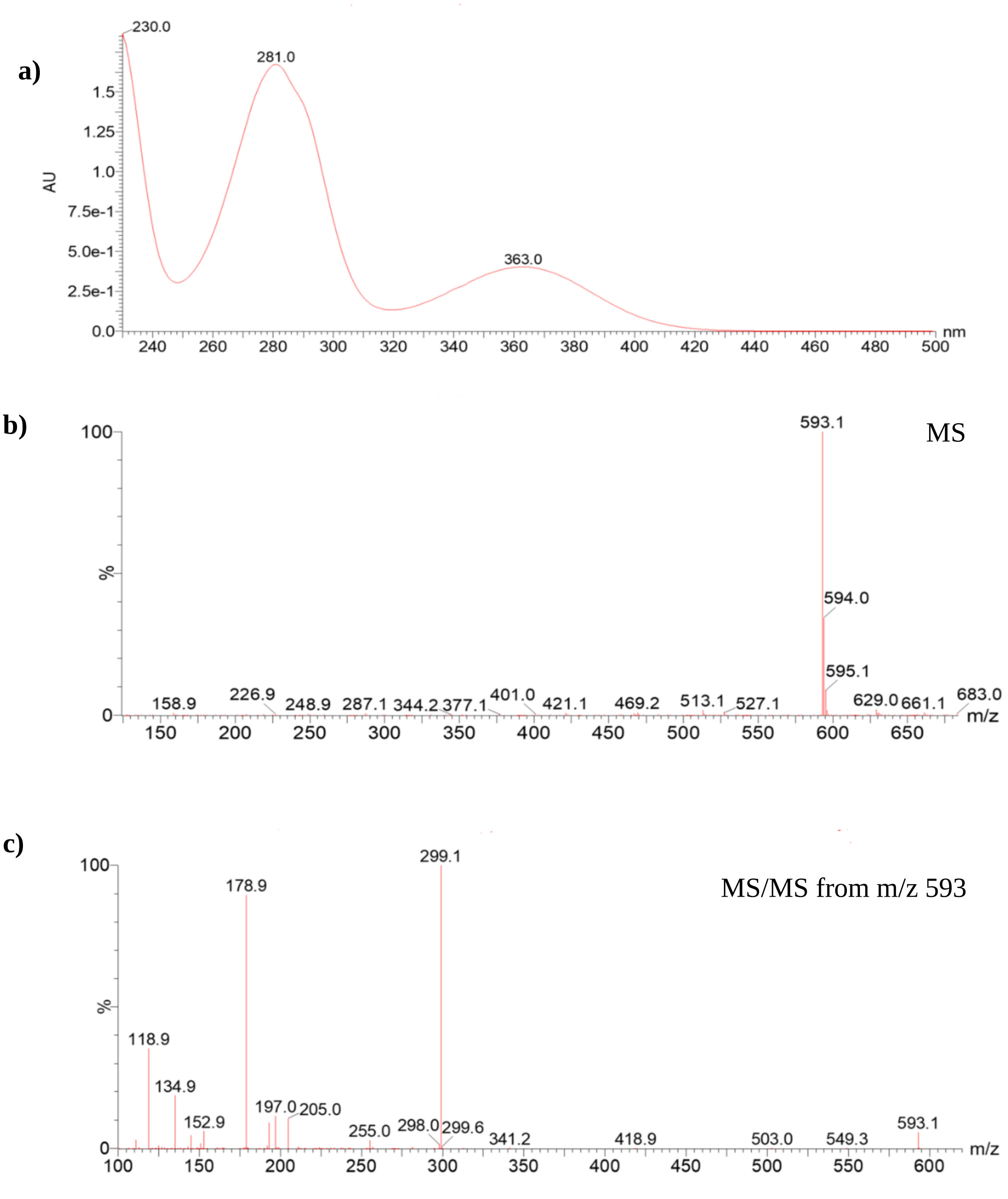

Figure S6: UHPLC-UV/MS/MS data of peak 12. a) UV-Vis spectrum; b) full scan negative ion electrospray mass spectrum ; c) negative ion electrospray MS/MS spectrum, with collision energy of 40V. This matched miconioside B.

a)

b)

c)

c)

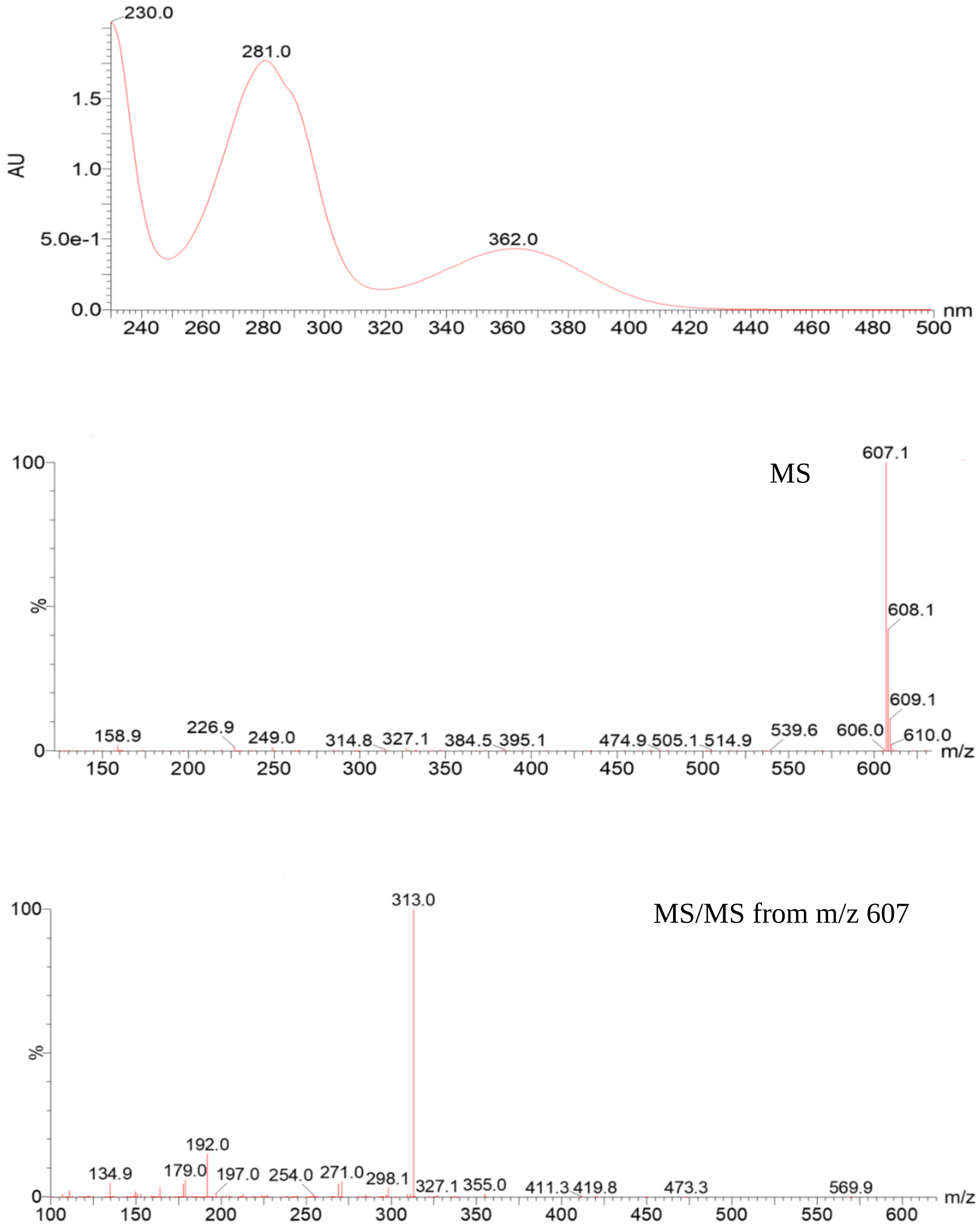

Figure S7: UHPLC-UV/MS/MS data of peak 13. a) UV-Vis spectrum; b) full scan negative ion electrospray mass spectrum ; c) negative ion electrospray MS/MS spectrum, with collision energy of 40V. This matched matteucinol 7-*O*-β-apiofuranosyl (1 → 6)-β-glucopyranoside.

a)

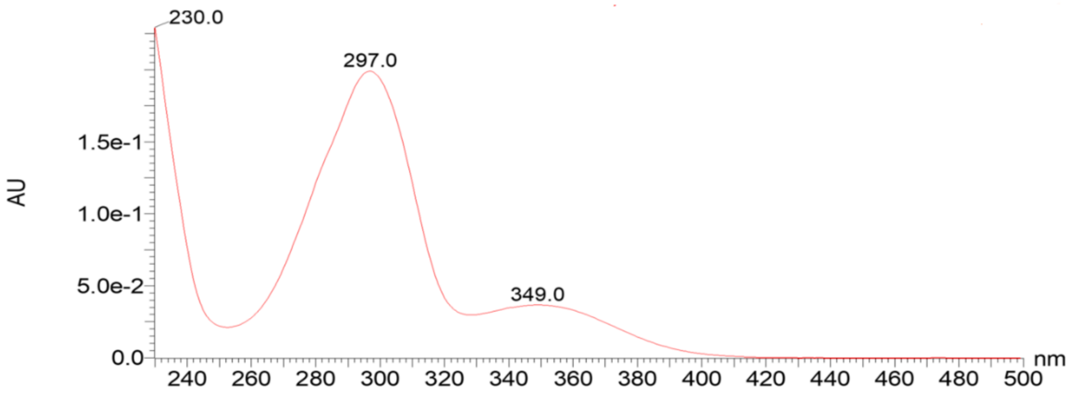

b)

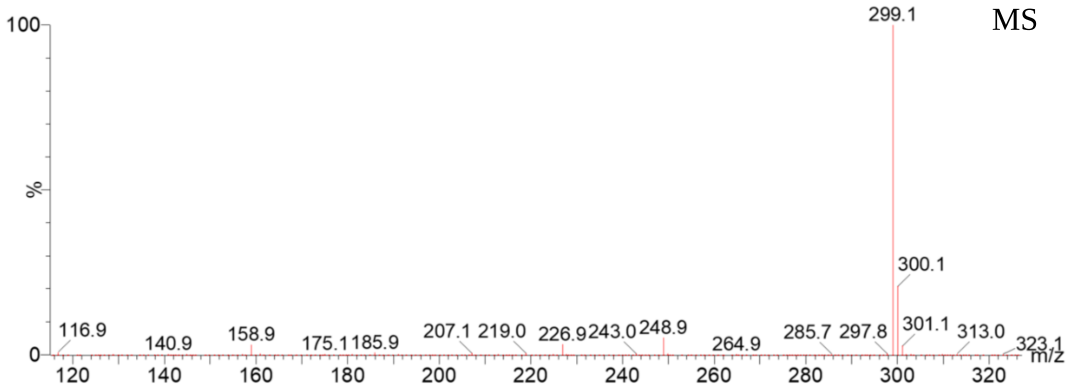

c)

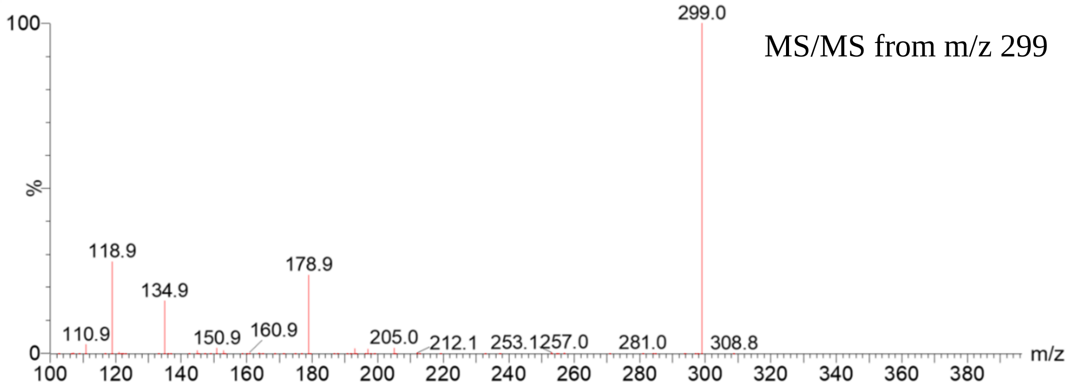

Figure S8: UHPLC-UV/MS/MS data of peak 14. a) UV-Vis spectrum; b) full scan negative ion electrospray mass spectrum c) negative ion electrospray MS/MS spectrum, with collision energy of 40V. This matched farrerol.

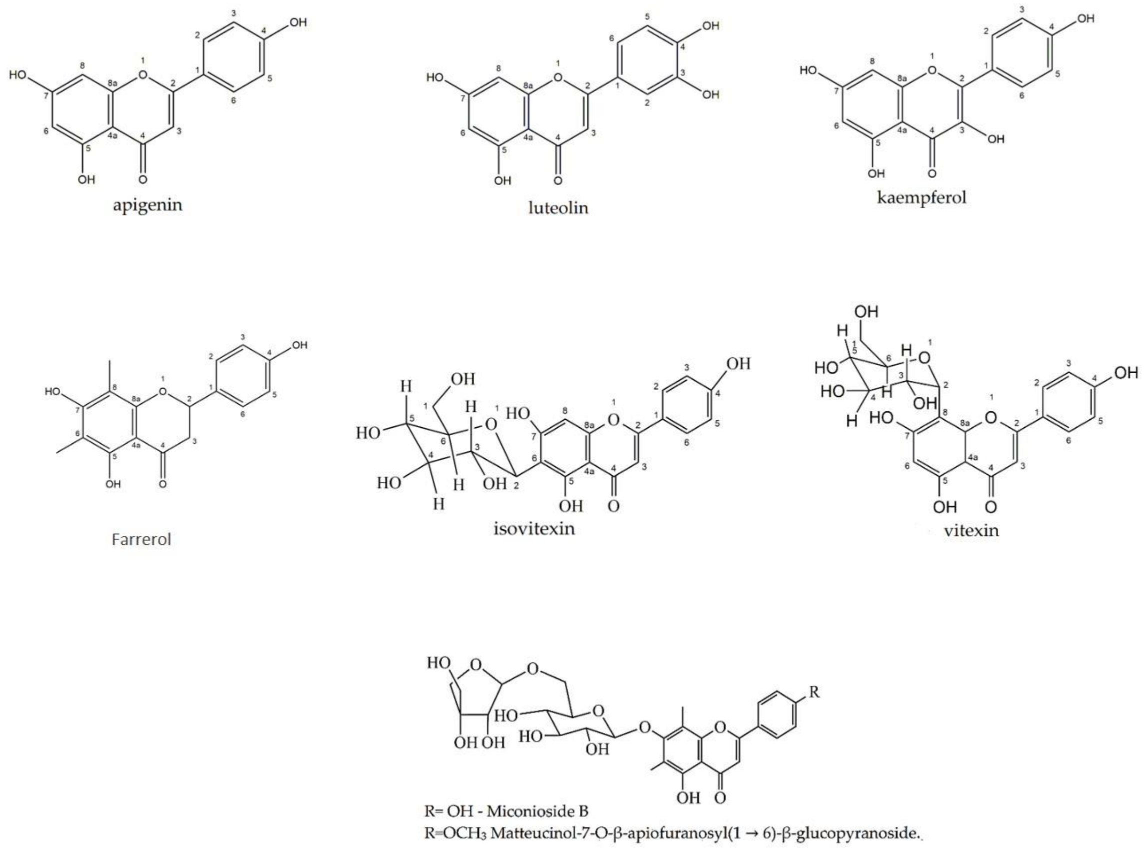

Figure S9: Chemical structures of the flavonoids from *M. chamissois* Naudin aqueous extract from leaves (AEMC).

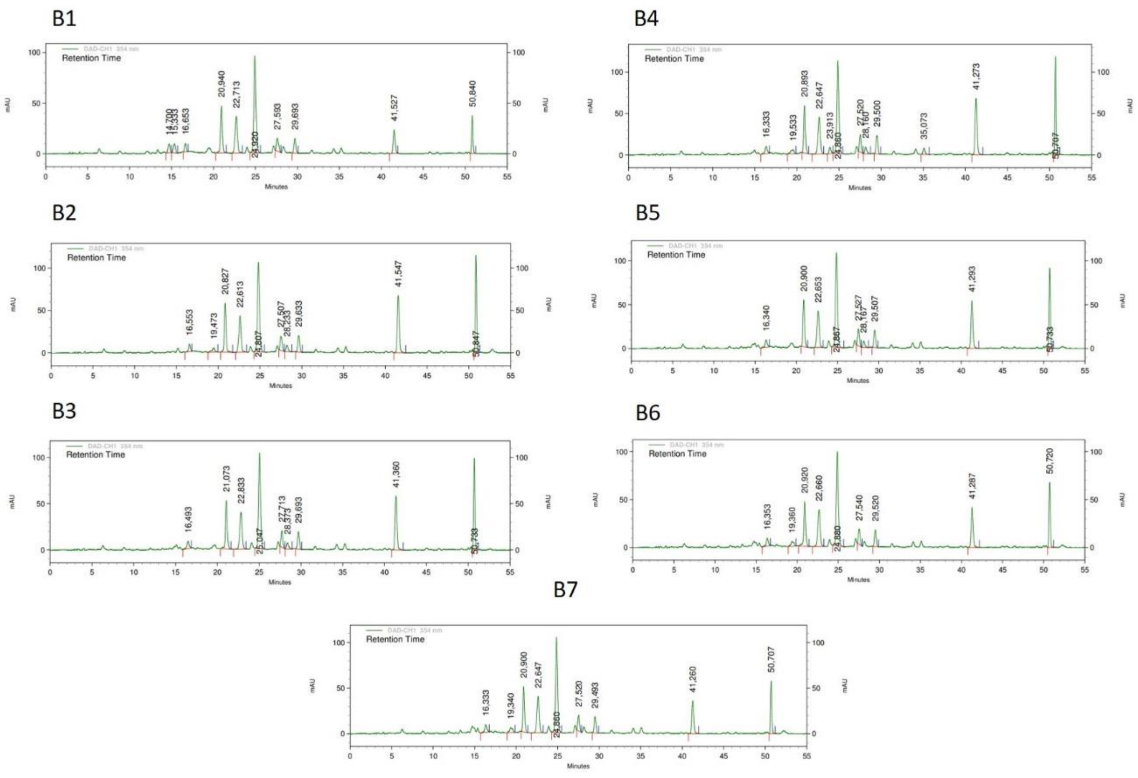

Figure S10: Chromatographic profile of aqueous extract *M. chamissois* Naudin leaves in different batches (B1-B7) by HPLC/DAD at 354 nm. Detection at 354 nm, C18 column, flow rate of 0.6 mL/min, eluent: phosphoric acid 1%, and acetonitrile in gradient system.
